# Accumulation of natural killer cells in ischemic brain tissues and the chemotactic effect of IP-10

**DOI:** 10.1186/1742-2094-11-79

**Published:** 2014-04-17

**Authors:** Yao Zhang, Zhongming Gao, Dandan Wang, Tongshuai Zhang, Bo Sun, Lili Mu, Jinghua Wang, Yumei Liu, Qingfei Kong, Xijun Liu, Yue Zhang, Haoqiang Zhang, Jiqing He, Hulun Li, Guangyou Wang

**Affiliations:** 1 Department of Neurobiology, Harbin Medical University Provincial Key Lab of Neurobiology, Harbin Medical University, Xuefu Road, 150081, Heilongjiang, People’s Republic of China; 2 Neurology department, The Affiliated Hospital of Hangzhou Normal University, Wenzhou Road, 310015 Zhejiang, People’s Republic of China; 3 The Key Laboratory of Myocardial Ischemia, Harbin Medical University, Ministry of Education, Xuefu Road, 150001, Heilongjiang, Province People’s Republic of China; 4 Key Lab of Magnetic Resonance Imaging Device and Technique, Huayuanbei Road, 100082 Beijing, People’s Republic of China; 5 Department of Otorhinolaryngology, Mudanjiang 2nd People’s Hospital, Guanghua Road, 157005, Heilongjiang, People’s Republic of China

**Keywords:** NK cells, Cerebral ischemia, IP-10, CXCR3

## Abstract

**Background:**

Stroke is accompanied by a distinguished inflammatory reaction that is initiated by the infiltration of immunocytes, expression of cytokines, and other inflammatory mediators. As natural killer cells (NK cells) are a type of cytotoxic lymphocyte critical to the innate immune system, we investigated the mechanism of NK cells-induced brain injuries after cerebral ischemia and the chemotactic effect of IP-10 simultaneously.

**Methods:**

NK cells infiltration, interferon-gamma (IFN-γ) and IP-10 expression were detected by immunohistochemistry, immunofluorescence, PCR and flow cytometry in human and C57/BL6 wild type mouse ischemic brain tissues. The ischemia area was detected via 2,3,5-triphenyltetrazolium chloride staining. CXCR3 mean fluorescence intensity of isolated NK cells was measured by flow cytometry. The neuronal injury made by NK cells was examined via apoptosis experiment. The chemotactic of IP-10 was detected by migration and permeability assays.

**Results:**

In human ischemic brain tissue, infiltrations of NK cells were observed and reached a peak at 2 to 5 days. In a permanent middle cerebral artery occlusion (pMCAO) model, infiltration of NK cells into the ischemic infarct region reached their highest levels 12 hours after ischemia. IFN-γ-positive NK cells and levels of the chemokine IP-10 were also detected within the ischemic region, from 6 hours up to 4 days after pMCAO was performed, and IFN-γ levels decreased after NK cells depletion *in vivo*. Co-culture experiments of neural cells with NK cells also showed that neural necrosis was induced via IFN-γ. In parallel experiments with IP-10, the presence of CXCR3 indicates that NK cells were affected by IP-10 via CXCR3, and the effect was dose-dependent. After IP-10 depletion *in vivo*, NK cells decreased. In migration assays and permeability experiments, disintegration of the blood–brain barrier (BBB) was observed following the addition of NK cells. Moreover, in the presence of IP-10 this injury was aggravated.

**Conclusions:**

All findings support the hypothesis that NK cells participate in cerebral ischemia and promote neural cells necrosis via IFN-γ. Moreover, IP-10 intensifies injury to the BBB by NK cells via CXCR3.

## Background

The immune system and the central nervous system (CNS) can affect one another both locally and systemically. For example, during a stroke, the immune response of the CNS induces activation of resident glia cells and macrophage [1-4], as well as the infiltration of circulating immune cells, respectively. A stroke is hypothesized to be a multiphasic process [5] and, in most cases, the cause of death results from inflammation without exogenous infection. Thus, a secondary component of ischemic brain injury may involve the effects of a significant inflammatory response.

In the first hours following stroke, rapid activation of resident microglia and production of pro-inflammatory cytokines occurs [6]. In addition, neutrophils and monocytes and/or macrophage infiltrate and accumulate in microvessels and in the ischemic cerebral parenchyma [7]. Accumulating evidence further suggests that the resulting inflammation response is responsible for inducing cerebral injury that is secondary to the hypoxia and ischemia that predominantly characterize a stroke event [8]. However, it is important to note that the action of the immune system is not uniform, and activation of immune cells may play different roles at different times.

Natural killer (NK) cells are a type of cytotoxic lymphocytes that play an important role in the innate immune system. For example, NK cells mediate a response to virally infected cells within three days of infection, and respond to tumor formation. The term ‘natural killers’, was coined based on the initial notion that these lymphocytes do not need to be activated to kill cells [9]. NK cells are key components of the innate immune system since they are able to rapidly produce an abundance of cytokines, mainly IFN-γ, and are able to lyse target cells without prior sensitization [10,11]. The role of NK cells in the development of adaptive immune responses has also recently been shown [12].

Numerous observations have suggested that NK cells play a role in the adaptive immune response associated with autoimmune diseases. For example, the expansion of blood NK cells has been found to correlate with the suppression of disease activity, and NK cells isolated from patients during treatment were found to be highly activated [13-15].

While stroke is a disease that directly affects the neural system, it is also a type of autoimmune disease involving non-infection induced inflammation. Peterfalvi *et al.*[16] proved that NK cells and its fundamental cytokines decrease after stoke in human peripheral circulation. As cerebral ischemia is a kind of focal disease, focusing on changes in central system becomes necessary. Therefore, the objective of this study was to investigate the possible role of NK cells in stroke, including the timing of their infiltration into the brain, whether they mediate beneficial or harmful effects, what kind of role they may have in cerebral ischemia, and what kind of changes NK cells undergo during this process. Accordingly, functional alterations in cytokine and chemokine production by NK cells in both human and mouse models during the acute phase of ischemic stroke were examined.

## Methods

### Animal experiments

Adult male C57/BL6 mice (weighing 20 to 25 g) were obtained from Peking Vital River Laboratory Animal Ltd (Beijing, People’s Republic of China). All mice were bred and maintained in accordance with the guidelines of the Care and Use of Laboratory Animals published by the China National Institute of Health. Furthermore, all experiments were conducted in accordance with institutional guidelines and were approved by Harbin Medical University ethics committee. A permanent middle cerebral artery occlusion (pMCAO) mouse model was established using the intraluminal filament method (6–0 nylon) as previously described [17]. In the sham group arteries were blunt dissected, yet not ligated. Following surgery, each mouse was assessed on a scale from 0 to 5 upon awakening, and only mice receiving a score of > 1 were included in this study [18].

### Immunohistochemistry and immunofluorescence staining for NKp46, IP-10, CXCR3 and IFN-γ

Human samples were provided by the Department of Neurosurgery, The First Clinical College of Harbin Medical University and were used for our research which was approved by the Harbin Medical University ethics committee. Patient deaths were identified as due to cerebral ischemic infarction and were diagnosed using computerized tomography. Tissue samples were taken from an area adjacent to the infarct site, and also from a similar area on the normal contralateral hemisphere, from each human brain sample. Tissues were embedded in paraffin and sectioned (10 μm). Frozen CNS material from mouse models were obtained after autopsy. Briefly, after being fixed in cold acetone for 20 minutes, tissues were blocked in 5% horse serum for 1 hour at room temprature, and were then permeabilized with 0.1% Triton X-100 (Sigma, St. Louis, USA) for 30 minutes. Primary antibodies used included: goat anti-mouse/human NKp46 (1/150, Santa Cruz, Dallas, USA), goat anti-mouse/human IP-10 (1/150, Santa Cruz, Dallas, USA), rabbit anti-mouse CXCR3 (1/150, Santa Cruz, Dallas, USA), and rat anti-mouse IFN-γ (1/150, Biolegend, San Diego, USA). Tissues were incubated with antibodies overnight at 4°C. After several washes with phosphate buffing saline (PBS), the appropriate secondary antibodies were applied (1:200, Zhongshan Goldenbridge Biotechnology Co. Ltd., Beijing, People’s Republic of China). Nuclei were co-stained with 4,6-diamidino-2-phenylindole (DAPI). Control stainings included the omission of primary antibody. The human tissue section areas were measured by image analysis software (Image Pro Plus 5.0, Media Cybernetics, Warrendale, USA). The numbers of stained cells per mm^2^ of tissue area were calculated.

### 2,3,5-triphenyltetrazolium chloride staining

Mice were euthanized 12 hours after pMCAO under deep anesthesia with sodium pentobarbital (60 mg/kg body weight). Brains were rapidly removed after intracardial infusion of PBS. The brains were then cut on the coronal suture into five slices, using a brain slicer. Brain slices were immediately incubated in 2,3,5-triphenyltetrazolium chloride (TTC) solution (2% solution in PBS) for 30 minutes at 37°C. Areas not stained red with TTC were considered to be damaged.

### Primary cultures of neural cells

Neural cell cultures were prepared from newborn C57/BL6 mice. Soon after discarding the meninges, the mesencephalon, cerebellum, and brain stem, cortexes were blunt isolated from the cerebrum and then gently pipetted to achieve a single cell suspension in 10% DMEM (supplemented with 10% FCS, 100 U/ml penicillin, 0.1 mg/ml streptomycin). Cell suspensions were passed through a 150 μm nylon filter, then were seeded in 24-well plates (5 × 10^4^ cells/well) or 25 mm^2^ flasks (10^5^/ml) coated with D-polylysine (PDL), and were cultured for at least 7 days.

### Flow cytometry

Mice were euthanized and perfused with PBS. Hemispheres were divided to isolate non-ischemic (ipsilesional) and ischemic (contralesional) tissue samples. Three to four hemispheres were pooled for each treatment group, and the resulting homogenates were pressed through 150 μm and 75 μm filters. Next, cells were separated from myelin and debris using 70% and 30% Percoll (GE Healthcare, Pittsburgh, USA) gradients, respectively, followed by centrifugation. Samples were then incubated with anti-mouse NKp46 antibody (Santa Cruz, Dallas, USA) for 30 minutes at RT in a fluorescence-activated cell sorter (FACS) buffer (0.1% bovine serum albumin (BSA), 0.01% sodium azide in PBS). After the cells were washed twice with wash buffer (0.1% BSA/PBS), FITC-conjugated anti-goat secondary antibodies (Biolegend, San Diego, USA) were applied for 30 minutes at RT. Finally, the cells were fixed with 2% paraformaldehyde at 4°C, and were then analyzed by blinded evaluators using a FacsCalibur (BD Biosciences, Franklin Lakes, USA) and FlowJo software (ThreeStar Inc., Ashland, USA) Three independent experiments were performed for all time points, and 3 to 4 mice were used per experiment.

Neural cells for apoptosis detection were prepared from newborn C57/BL6 mice as previously described, and were cultured in 10% DMEM until they covered the bottom of a 25 mm^2^ flask. Cells were then treated with NK cells (10^5^) harvested from C57/BL6 12 weeks mice’ spleens with a MagCellect Mouse NK Cell Isolation Kit (R&D, Minneapolis, USA) and murine IFN-γ neutralizing antibody (10 ng/ml, R&D, Minneapolis, USA) in 10% DMEM, then stained with a PI/Annexin kit (BD Biosciences, Franklin Lakes, USA). Stained cells were detected by evaluators who have no relationship with this experiment using a FacsCalibur (BD Biosciences, Franklin Lakes, USA) and FlowJo software. Three independent experiments were performed.

NK cells harvested from 12-week old C57/BL6 mice spleens with a MagCellect Mouse NK Cell Isolation Kit (R&D, Minneapolis, USA) were treated with murine IP-10 (10 ng/ml, 50 ng/ml, 100 ng/ml) accompanied by 6 hour oxygen glucose deprivation (OGD) in Hank’s Balanced Salt Solution (HBSS) (Ca-Mg-free, glucose 1 g/L). Mean fluorescence of CXCR3 (PE) which NK cells presented were blinded evaluators using a FacsCalibur (BD Biosciences, Franklin Lakes, USA) and FlowJo software. Three independent experiments were performed. Non-OGD groups were cultured in a cell incubator (5%CO_2_) in HBSS (Ca-Mg-free, 1 g/L glucose). Three independent experiments were performed.

### Reverse transcription PCR

Total RNA from the whole cortex, which had been sorted in pMCAO mice, was obtained at different time points using a Trizol extraction method recommended by Invitrogen. Reverse transcription was performed using an RT-PCR kit from TaKaRa (Kusatsu, Japan). PCR amplification of IP-10,IFN-γ, and glyceraldehyde-3-phosphate dehydrogenase (GAPDH) (endogenous control) were achieved using Taq polymerase and the following primers: IP-10 sense 5′-GCC GTC ATT TTC TGC CTC AT-3′ and anti-sense 5′-GCT TCC CTA TGG CCC TCA TT-3′, IFN-γ sense 5′-AGC GGC TGA CTG AAC TCA GAT TGT-3′ and anti-sense 5′-GTC ACA GTT TTC AGC TGT ATA GGG-3′, and GAPDH sense 5′-AAT GCA TCC TGC ACC AA-3′ and anti-sense 5′-TCC ACC ACC CTG TTG CTG TA-3′. A total of 40, 40 and 32 cycles were performed for IP-10, IFN-γ and GAPDH, respectively. Three independent experiments were performed.

### Migration assays

Neural cells (5 × 10^4^ cells) prepared from newborn C57/BL6 mice as previously described were seeded in the bottom of 24-well transwell plates. After culturing the cells for at least 7 days, mouse brain endothelial cells (bEnd3, ADCC) (1 × 10^4^) were plated on top of 2% gelatin-coated 3 μm pore size upper transwell chambers with 10% DMEM media. After the ECs formed a confluent monolayer, 20 ng/ml murine IP-10 neutralization antibody (R&D, Minneapolis, USA) was added to the lower chambers when appropriate. Simultaneously, a suspension of NK cells (1 × 10^6^/ml; 100 μl) prepared from 12-week old C57/BL6 mice spleens with a MagCellect Mouse NK Cell Isolation Kit (R&D, Minneapolis, USA) were added to the upper chamber. The ability of NK cells to cross the monolayer was evaluated by counting the absolute number of cells that migrated to the lower chamber after OGD conditions were applied for 6 hours in HBSS (Ca-Mg-free, 1 g/L glucose). Furthermore, the cells in the upper chamber were indirectly labeled with NKp46-FITC, cell counts were performed using FACS, and three independent experiments were performed. Non-OGD groups were cultured in a cell incubator (5%CO_2_) in HBSS (Ca-Mg-free, 1 g/L glucose).

### Permeability assays

Permeability assays were conducted as previously described [19]. Soon after 6 hours OGD in HBSS (Ca-Mg-free, 1 g/L glucose), 50 μg/ml fluorescein isothiocyanate-labeled BSA (FITC-BSA, Sigma, St. Louis, USA) was added to the upper chamber of transwells. Samples (both 50 μl) were then obtained from the upper and lower chambers of each well and the fluorescence intensity of these samples was measured using a FL600 microplate fluorescent reader (Biotek, Vermont, USA). The diffusion rate, representing the permeability of blood brain barrier (BBB) endothelial cells (ECs), was expressed as a percentage and was calculated as follows: (BSA lower chamber) × 100 / (BSA upper chamber). Non-OGD groups were cultured in a cell incubator (5%CO_2_) in HBSS (Ca-Mg-free, 1 g/L glucose).Three independent experiments were performed.

### *In vivo* NK depletion assay

Depletion of NK cells *in vivo* in mice was induced by intraperitoneal injection (i.p.) of a depleting anti-NK1.1 mAb (clone PK136, Biolegend, San Diego, USA). The depleting antibody was given at 0.5 mg i.p. 1 day before the pMCAO mouse model (12 hours) was established as previously described. The lymphocytes infiltration assay was then performed via FACS, and IFN-γ expression level was detected by RT-PCR as previously described.

### *In vivo* IP-10 depletion assay

To block IP-10 *in vivo*, mice were injected intravenously with 3 mg/100 g body weight anti-mouse IP-10 (R&D Systems) for two days to neutralize the IP-10 secretion before the pMCAO mouse model was established as previously described, and the lymphocytes infiltration assay was then performed via FACS.

### Statistical analysis

Results are expressed as the mean ± standard deviation (SD). An analysis of variance (ANOVA) test was used to compare multiple quantitative variables. Statistical analysis was performed using one-way ANOVA analysis of variance followed by Student’s t-test. Statistical significance was considered to be *P* < 0.05.

## Results

### NK cells participate in cerebral ischemia

**Figure 1:**
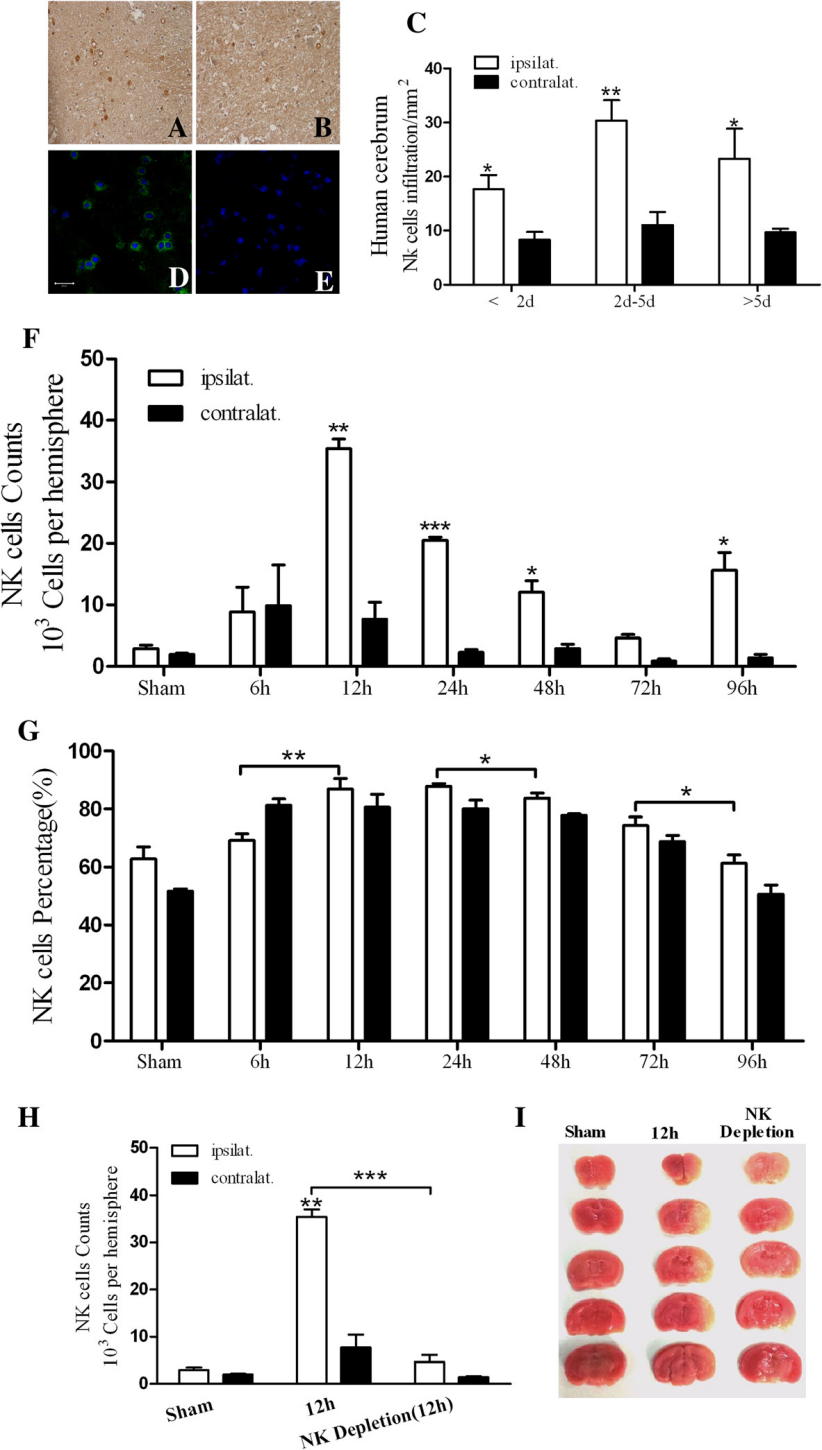
**NK cells participate in cerebral ischemia. (A,B)**: NK cells stained brown in ischemic tissues (magnification, 400×). Differences between the ischemic **(A)** and non-ischemic **(B)** hemispheres were significant for the 2 to 5 day group, and were fewer in the non-ischemic hemispheres; **(C)**: Infiltration of NK cells into human ischemic tissue. The highest number of NK cells were detected in the 2 to 5 day group; **(D,E)**: NK cells in mouse ischemic tissue penumbra. NK positive cells (FITC) were found in ischemic tissues **(D)** (bar = 30 μm); **(F)**: Infiltration of NK cells detected in mouse ischemic tissue by FACS; **(G)**: Of the infiltrating lymphocytes detected, the percentage of NK cells is reported; **(H)**: After NK cells depletion *in vivo*, infiltration of NK cells detected in mouse ischemic tissue by FACS; **(I)**: Representative TTC-stained sections of NK cells depletion mouse at 12 hours following pMCAO. (*P < 0.05, **P < 0.01, ***P < 0.001, compared with the corresponding values for the contralateral hemispheres). pMCAO, permanent middle cerebral artery occlusion.

Paraffin-embedded human brain tissues affected by cerebral ischemic infarction, as well as contralateral tissues, were divided into three groups according to the period of time that elapsed between the onset of stroke and death. These groups included: <2 days, 2 to 5 days, and >5 days, with 5, 11, and 4 sets of patient tissues included in each group, respectively (Table 1, 1–20). Immunohistochemistry staining of NK cells was performed for each tissue set (Figure 1A,B), and the number of NK cells in the ischemic hemispheres was found to be significantly greater than those in the non-ischemic hemispheres (*P*_< 2 days_ < 0.05, *P*_2 to 5 days_ < 0.01, *P*_*>*5 days_ < 0.05). Furthermore, the highest numbers of NK cells were observed in the 2 to 5 days tissues (Figure 1C).

**Table 1:** Clinical data from stroke patients

**Number**	**Serial number**	**Gender**	**Age**	**Diagnosis**	**First or recurrent cerebrovascular accident**	**Medications**
1	A223	Male	60	Ischemic stroke	First	Mannitol, Lasix, buffered glycerol saline, Coraminum, lobeline, glucose, insulin, mefoxinvial, albumin, urokinase
2	A593	Female	65	Ischemic stroke	Recurrent	Mannitol, Xuesaitong injection, CDPC, urokinase, Dextran, insulin, cephalosporin, Coraminum, lobeline
3	A437	Female	48	Ischemic stroke	First	Mannitol, Lasix, buffered glycerol saline, Coraminum, lobeline, glucose, insulin, mefoxin vial
4	A225	Female	63	Ischemic stroke	Recurrent	mannitol, Lasix, Dexamethasone, Saline, Sodium Valproate
5	A351	Female	44	Ischemic stroke	First	Saline, ahylysantinfarctase
6	A368	Male	65	Ischemic stroke	First	mannitol, Lasix, Coraminum, lobeline
7	A375	Male	60	Hemorrhagic stroke	First	mannitol , PAMBA, Lasix, cedilanid
8	A388	Female	56	Ischemic stroke	First	Mannitol, Lasix, buffered glycerol saline, urokinase, glucose, cephalosporin, Dextran, CompoundDanshen, Nao Mai Tong recipe, Coraminum, lobeline
9	A389	Female	68	Ischemic stroke	First	Mannitol, Lasix, buffered glycerol saline, Coraminum, lobeline
10	A405	Female	56	Ischemic stroke	First	Mannitol, Lasix, buffered glycerol saline, Sodiumcytidine diphosphate choline injection, cephalosporin, Coraminum, lobeline, insulin
11	A422	Male	43	Ischemic stroke	First	cidilaniol, glucose, injcinetodini, coramini, lobeline, Saline, dopamim, aramim, ritalim, atropiai, epinephrine, sodium bicarbonate, noradrenaline
12	A432	Male	64	Ischemic stroke	Recurrent	Saline, ahylysantinfarctase, mannitol, Lasix, Coraminum, glucose, injcinetodini, coramini, lobeline, Saline, dopamim, aramim, ritalim, atropiai, epinephrine, sodium bicarbonate, noradrenaline
13	A597	Male	64	Ischemic stroke	Recurrent	Mannitol , Xuesaitong injection, CDPC, urokinase, Dextran, insulin, cephalosporin, Coraminum, lobeline
14	A598	Male	48	Ischemic stroke	First	Mannitol, Lasix, buffered glycerol saline, Coraminum, lobeline, glucose, insulin, mefoxin vial
15	A599	Male	67	Ischemic stroke	Recurrent	Mannitol, Lasix, buffered glycerol saline, Coraminum, lobeline, glucose, mefoxinvial, albumin
16	A600	Female	61	Ischemic stroke	First	Mannitol, Lasix, buffered glycerol saline, Coraminum, lobeline, glucose, insulin, mefoxinvial, albumin, urokinase
17	A601	Male	67	Ischemic stroke	First	Mannitol, Lasix, buffered glycerol saline, Coraminum, lobeline, glucose, mefoxinvial, urokinase, Losec, Cimetidine
18	A602	Male	67	Ischemic stroke	First	Mannitol, Lasix, Nimotop, Aspirin,
19	A610	Male	63	Ischemic stroke	Recurrent	Insulin, glucose,
20	A625	Female	63	Ischemic stroke	First	Mannitol, Lasix, buffered glycerol saline, Coraminum, lobeline, glucose, insulin, mefoxinvial, albumin, urokinase
21	A634	Female	70	Ischemic stroke	First	Mannitol, Lasix, urokinase, Nitroprusside, insulin, insulin, dopamim
22	A647	Male	60	Ischemic stroke	First	Nimotop, Losec, Coraminum, lobeline, Citicoline, mannitol
23	A648	Female	47	Ischemic stroke	First	Lasix, Luminal, urokinase, insulin, glucose, KCl, NaCl
24	A655	Male	66	Ischemic stroke	Recurrent	Saline, ahylysantinfarctase, mannitol, Lasix, Coraminum, glucose, injcinetodini, coramini, lobeline, Saline, dopamim, ritalim, atropiai, sodium bicarbonate, noradrenaline
25	6571	Female	53	Ischemic stroke	First	Mannitol, Lasix, buffered glycerol saline, urokinase, glucose, cephalosporin, Dextran, Coraminum, lobeline
26	6572	Female	56	Ischemic stroke	First	Mannitol, Lasix, buffered glycerol saline, urokinase, glucose, cephalosporin, Dextran, CompoundDanshen, Coraminum, lobeline
27	6573	Female	73	Ischemic stroke	First	Mannitol, Lasix, urokinase, Nitroprusside, dopamim
28	6574	Male	62	Ischemic stroke	Recurrent	Mannitol, Lasix, urokinase, insulin, glucose,
29	6575	Male	59	Hemorrhagic stroke	First	Mannitol, PAMBA, Lasix, cedilanid
30	6576	Male	66	Ischemic stroke	Recurrent	Mannitol, Xuesaitong injection, urokinase, Dextran, insulin, cephalosporin, Coraminum, lobeline
31	6577	Female	49	Ischemic stroke	First	Mannitol, Lasix, buffered glycerol saline, Coraminum, lobeline, glucose, insulin
32	6578	Male	64	Ischemic stroke	Recurrent	Mannitol, Lasix, buffered glycerol saline, insulin, glucose
33	6579	Female	45	Ischemic stroke	First	Mannitol, Lasix, buffered glycerol saline, Coraminum, lobeline, glucose, insulin, mefoxin vial

Next, the infiltration of NK cells was examined in a C57/BL6 pMCAO model. NK cells were detected in ischemic brain tissues, and these cells were consistently observed in proximity of the ischemic penumbra (Figure 1D,E). To monitor NK cells during ischemia, infiltrating cells were isolated from the cerebrum at various timepoints and were analyzed by flow cytometry. Based on the FACS data obtained, the infiltration of NK cells peaked 12 hours after ischemia, and the number of NK cells in the ischemic hemisphere was significantly greater than that of the non-ischemic hemisphere 12, 24, 48, and 96 hours after ischemia (*P*_12 hours_ < 0.01, *P*_24 hours_ < 0.001, *P*_48 hours_ < 0.05, *P*_96 hours_ < 0.05) (Figure 1F). Moreover, the percentage of NK cells among the infiltrating cells recovered was highest 12 hours after ischemia, and decreased at the subsequent timepoints (Figure 1G). After NK cells depletion, NK cells infiltration notably decrease in ischemic mouse brain at 12 hours (Figure 1H, *P* < 0.001), and the ischemia area diminished with TTC staining (Figure 1I).

### NK cells negatively affect neural cells via IFN**-γ**

**Figure 2:**
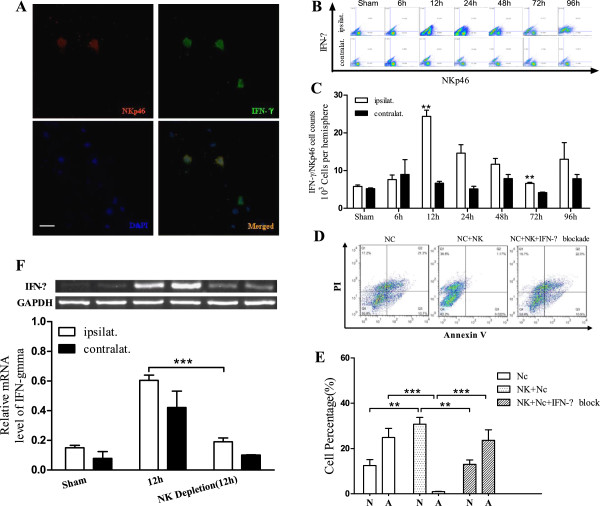
**NK cells promote neural necrosis via IFN-γ. (A)**: NK cells express IFN-γ in mouse ischemic cerebral tissue (bar = 20 μm); **(B,C)**: Infiltration of IFN-γ/NK positive cells in mouse ischemic brain detected by FACS at different time points; **(D,E)**: Percentage of neuronal cells undergoing apoptosis (A) and necrosis (N) after NK cells were co-cultured with or without IFN-γ; **(F)**: Levels of IFN-γ mRNA detected in mouse ischemic hemispheres by PCR after NK cells depletion *in vivo*; (**P < 0.01, ***P < 0.001, compared with the corresponding values of the contralateral hemispheres).

IFN-γ is one of the main cytokines secreted by NK cells. Using immunofluorescence assays and FACS, IFN-γ-positive NK cells present in ischemic tissues were analyzed 12, 24, 48, and 96 hours after ischemia (Figure 2A,B). The highest levels of IFN-γ-positive NK cells were detected 12 and 72 hours after ischemia (*P*_12 hours_ < 0.01, *P*_72 hours_ < 0.01) (Figure 2C).

Staining for apoptosis and necrosis, we found levels of necrosis of neural cells significantly increased after adding NK cells, compared with the control group (only neural cells), meanwhile levels of apoptosis were quite low(*P* < 0.001). However, after treatment with IFN-γ blockade, levels of apoptosis significantly increased and levels of necrosis became lower in the NK + IFN-γ blockade group, compared to the NK co-culture group (*P*_*N*_ < 0.01, *P*_*A*_ < 0.001), while levels of necrosis and apoptosis did not differ from the control group (Figure 2D,E). After NK cells depletion, the level of IFN-γ mRNA expression decreased at 12 hours (Figure 2F).

### Chemotactic effect of IP-10 on NK cells via CXCR3 during cerebral ischemia

**Figure 3:**
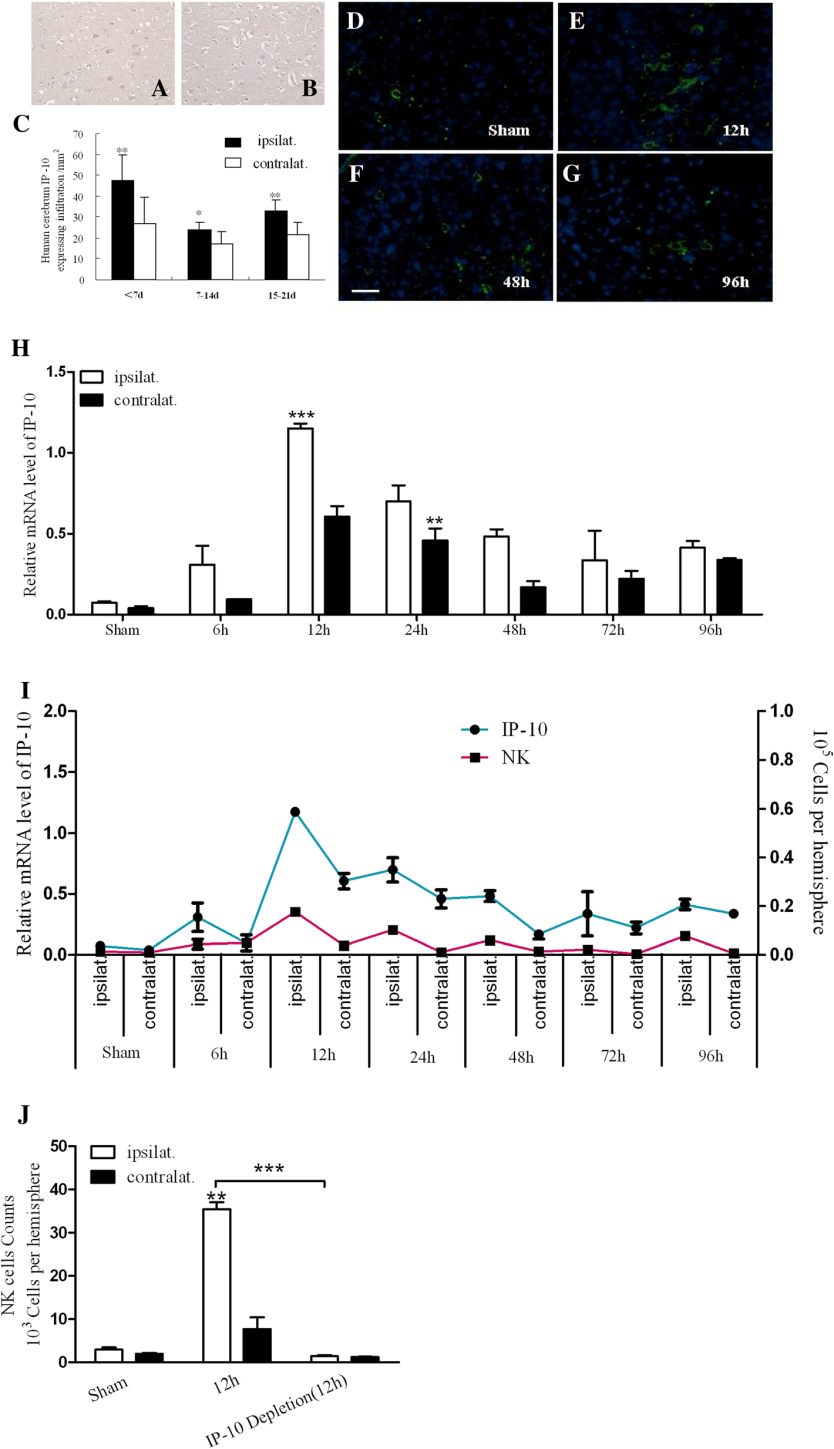
**Chemotactic effect of IP-10 on infiltrating NK cells during cerebral ischemia. (A,B)**: Expression of IP-10 in ipsilesional **(A)** and contralesional **(B)** hemispheres after stroke (magnification, 400×). **(C)**: Significant differences between ipsilesional and contralesional hemispheres were observed. **(D-G)**: Immunofluorescence detection of IP-10-positive cells (FITC) in mouse ischemic penumbra collected at different time points (bar = 20 μm). **(H)**: Levels of IP-10 mRNA detected in mouse ischemic hemispheres by PCR; **(I)**: Interaction between IP-10 and infiltrating NK cells; **(J)**: Infiltration of NK cells detected in mouse ischemic tissue by FACS after IP-10 depletion *in vivo*. (**P < 0.01, ***P < 0.001, compared with the corresponding values of the contralateral hemispheres).

A second set of human brain tissue samples were obtained, and these derived from patients that died <7 days, between 7 and 14 days, or >14 days after experiencing an ischemic event. There were four, five, and four tissue sets for each group, respectively (Table 1, 21–33). Immunohistochemistry assays were performed, including the staining of IP-10 (Figure 3A,B). A significantly higher number of IP-10-positive cells were detected in the ischemic hemispheres compared to the contralateral hemispheres among these three groups. In addition, IP-10-positive cells in the ischemic hemisphere tissues of the <7 day group were very high, and the IP-10-positive cells in the ischemic hemisphere tissues of the 7 to 14 days group were present at lower levels compared to the >14 day group (Figure 3C, *P*_<7day_ < 0.01, *P*_7 to 14 day_ < 0.05, *P*_15 to 21 day_ < 0.01).

Immunofluorescence assays were also performed for ischemic brain sections of pMCAO C57/BL6 mice. In these studies, IP-10 expressing cells were observed to localize to the ischemic penumbra, as well as to the sagittal suture, at different time points (Figure 3D,G). PCR assays demonstrated that levels of IP-10 mRNA were highest 12 hours after ischemia, especially in the ischemic hemisphere tissues (Figure 3H). Furthermore, the number of NK cells present in the ischemic and contralateral hemispheres of pMCAO mouse tissues was also detected by FACS, and a similar observation was made, with a greater number of NK cells detected in ischemic tissues than contralateral tissues. Taken together, the relationship between IP-10 levels and NK levels was found to be significant (Figure 3I, *P* < 0.001). Then we blocked IP-10 *in vivo* at the 12 hour time point, the number of NK cells present in the ischemic hemispheres of pMCAO mouse tissues were significantly low (*P* < 0.001) which were detected by FACS(Figure 3J, *P* < 0.001).

**Figure 4:**
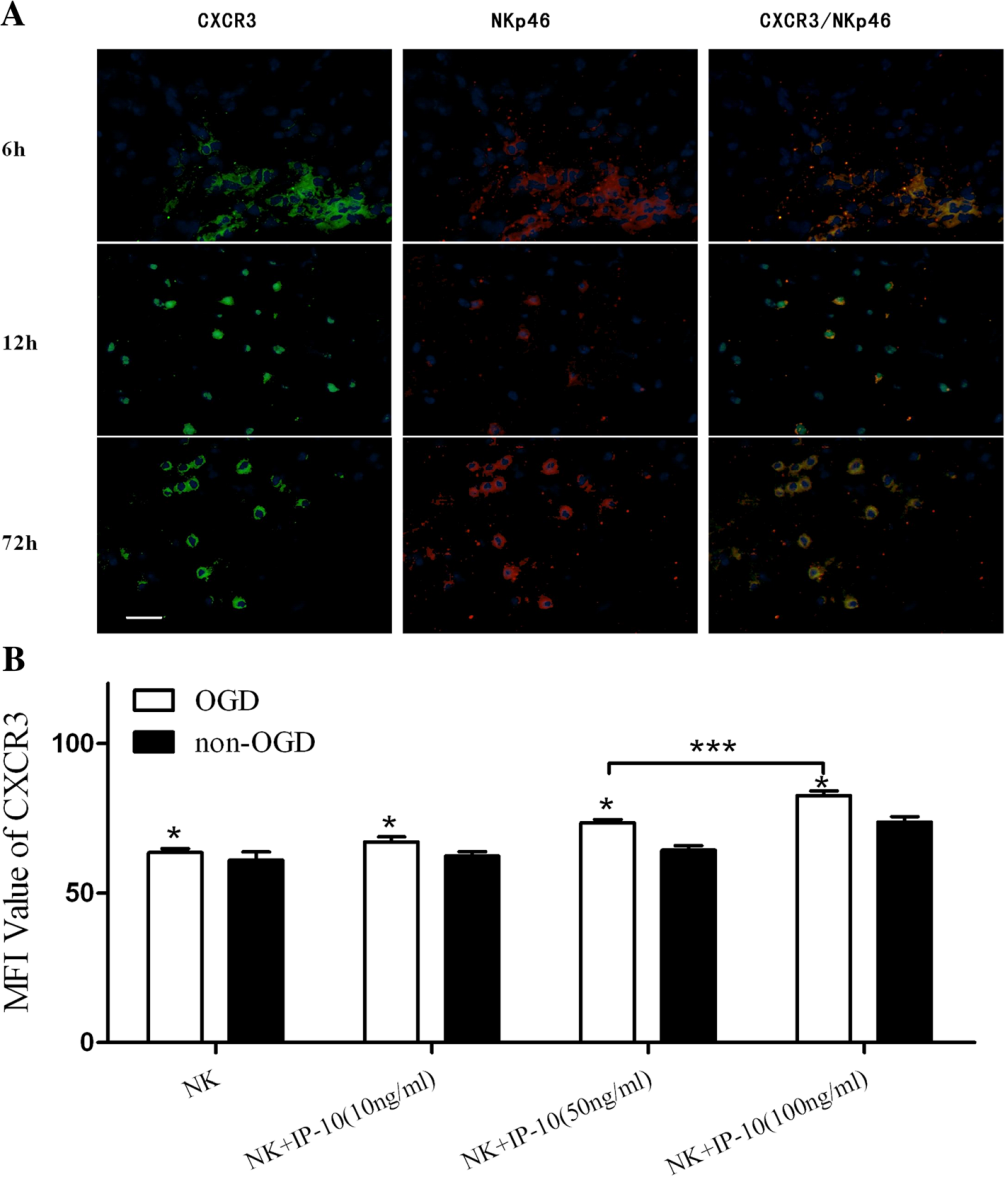
**NK cells were absorbed by IP-10 via the linkage of CXCR3. (A)** Immunofluorescence assays of NKp46 and CXCR3 in mouse ischemic cerebral tissue (bar = 20 μm); **(B)** The dose-dependent effect of IP-10 on NK cells. (*P < 0.05, ***P < 0.001).

In our laboratory, IP-10 has been found to be produced by local cells (glial cells and endothelial cells) in an inflammatory lesion and cells culture supernatants (data not shown). Therefore, expression of the receptor for IP-10, CXCR3, was assayed using immunohistochemistry. In mouse pMCAO brain tissue, CXCR3-positive infiltrating NK cells were detected (Figure 4A). NK cells were then treated with various concentrations of murine IP-10 (such as 10 ng/ml, 50 ng/ml, 100 ng/ml, Peprotech, USA) and subjected to 6 hour OGD conditions. Detection of the mean fluorescence value for CXCR3 indicated that CXCR3 expression in 100 ng/ml group significantly increased when compared with 50 ng/ml group (*P* < 0.001), and as the concentration of IP-10 increased, the mean fluorescence value of CXCR3 got higher. Furthermore, this number was enhanced when NK cells were deprived of oxygen (Figure 4B).

### NK cells accelerate BBB injury via IP-10 chemotaxis

**Figure 5:**
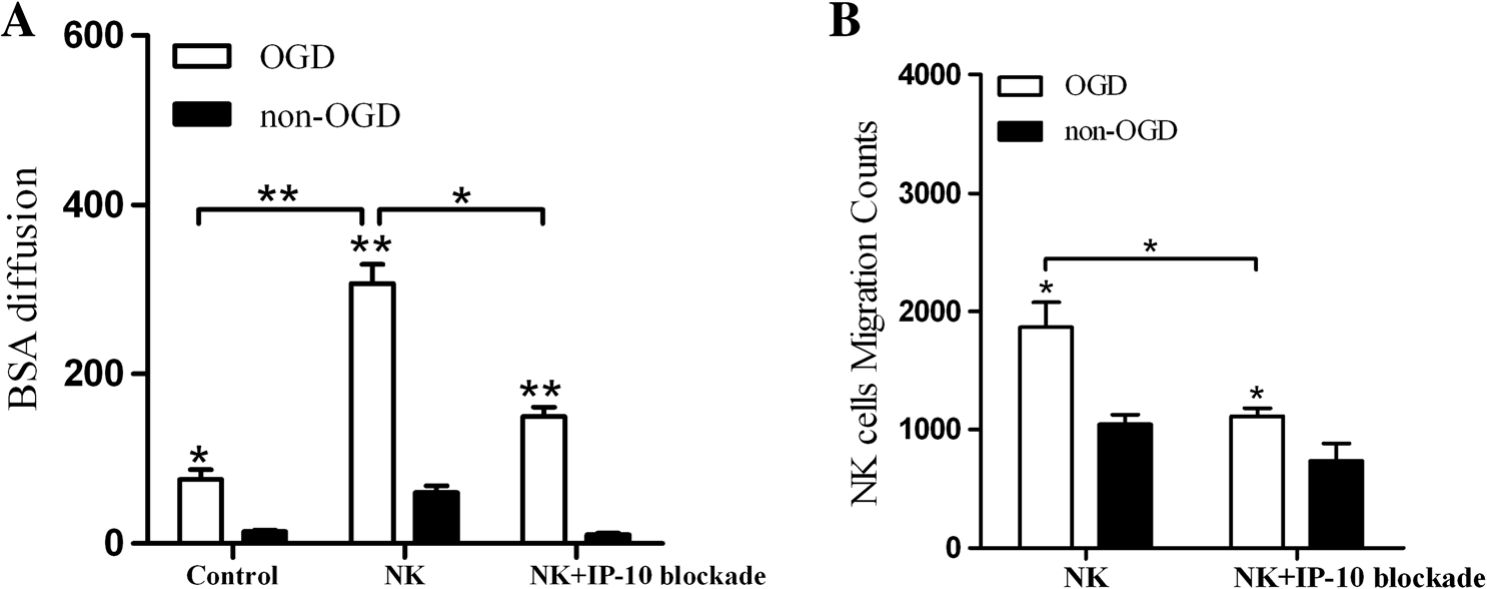
**IP-10 promotes NK cell-induced BBB injury. (A)**: IP-10 blockade attenuated a BBB permeability injury induced by NK cells under OGD treatment. **(B)**: Migration of NK cells with or without IP-10 blockade with OGD treatment. (*P < 0.05, **P < 0.01). BBB, blood brain barrier; OGD, oxygen glucose deprivation.

An *in vitro* model of the BBB was established with brain microvascular endothelial cells plated in the upper chambers of transwells, and neural cells plated in the lower chambers. Upon reaching confluence, the medium was changed to Ca-Mg-free HBSS (1 g/L glucose), and NK cells were added to the upper chamber. Cells were then subjected to OGD conditions. Using permeability assays, brain microvascular endothelial cells were found to be affected by the presence of NK cells after OGD (Figure 5A, *P* < 0.01), suggesting that NK cells not only negatively affect neural cells, but may also affect the BBB during ischemia.

To investigate the effect of IP-10 on NK cells, IP-10 antibody was added to the lower chamber of the BBB model to neutralize the IP-10 secreted by neural cells (results not shown). The permeability rate of this model subsequently decreased according to the BSA diffusion assay performed (Figure 5A, *P* < 0.05). FACS of neural cells in the lower chamber also detected a lower percentage of NK cells, thereby suggesting that the IP-10 blockade can inhibit the migration of NK cells through the BBB (Figure 5B, *P* < 0.05).

## Discussion

Peterfalvi *et al.*[16] previously demonstrated that NK cells are active during the acute phase of stroke. In addition, when NK cells were isolated from the peripheral circulation during the early phases of stroke, deficient IFN-γ production and cytotoxicity were observed, similar to that observed in animal models [20]. Additional reports have shown that while numbers of NK cells remain unchanged following stroke, numbers of adaptive T lymphocyte subsets decrease [21-23]. The objective of this study was to examine the role of NK cells in brain tissue affected by ischemia. During the early phases of stroke, a peak in the infiltration of NK cells was observed, concomitant with increased expression of IFN-γ (Figure 1A-C; Figure 2A-C). Based on these results, we hypothesized that NK cells play an important role during the onset of cerebral ischemia. In studies by Gelderblom *et al.*[24], NK cells were not found to significantly affect ischemia in a mouse brain 1 hour after the ischemic event and reperfusion. In the present study, numbers of infiltrating NK cells reached their highest levels in the ischemic hemisphere 12 hours after the ischemic event (*P <* 0.01) (Figure 1F) according to FACS analysis. Furthermore, IFN-γ-positive NK cells exhibited a similar profile (Figure 2C). The analysis of NK cells as a percentage of infiltrating lymphocytes further confirmed that NK cells have a predominant role during the onset of stroke (Figure 1G). After we depleted NK cells *in vivo*, NK infiltration in ischemic brain got notably lower (Figure 1H) as did the level of IFN-γ mRNA (Figure 2F), and the ischemia area diminished, which strengthens the importance of NK cells participation in the onset of cerebral ischemia.

Interestingly, when neural cells were exposed to activated NK cells, an increase in the number of dead cells and very low levels of apoptosis were observed. In contrast, a significant increase in the levels of apoptosis was detected following neutralization of IFN-γ secreted by NK cells (Figure 2D,E). Apoptosis is currently recognized as a necessary process during the development of the nervous system [25]. Moreover, the apoptosis of neural cells is anticipated to stabilize a microenvironment more so than the presence of dead neural cells. Therefore, we hypothesize that NK cells promote the necrosis of neural cells via IFN-γ during the early stages of stroke, and the fragments of these dead cells negatively affect the ischemic region. Accordingly, neutralization of IFN-γ in the brain should be further investigated in clinical trials.

The detection of NK cells in ischemic brain tissue illustrates that NK cells have a role in cerebral ischemia, and the section of IFN-γ by NK cells has the potential to affect neural cells. However, previous studies have not demonstrated how NK cells are able to access ischemic tissues. Since blood flow is blocked following an ischemic event, access via circulation is not a possibility. IP-10 is a chemoattractant secreted by several cell types in response to IFN-γ, and these cell types include monocytes, endothelial cells, and fibroblasts [26]. Furthermore, IP-10 has been identified as a chemoattractant for monocytes and macrophages, T cells, NK cells, and dendritic cells [27]. IP-10 has been proven to increase in focal stroke [28-30]. In our study, we focused on probing the relationship between IP-10 and NK cells accumulation after stroke. IP-10 was detected in human ischemic brain tissue (Figure 3A,B), and was present at high levels during the early stages of stroke (Figure 3C). Similarly, IP-10 was detected in ischemic cerebral tissue of the mouse model examined (Figure 3D,G), with the highest levels detected 12 hours after ischemia using RT-PCR. These results are consistent with the rat model of ischemia characterized by Wang *et al.*[31] (Figure 3H), and the decrease of NK cells infiltration in ischemic brain after IP-10 depletion *in vivo* prompt us to compare relationship between IP-10 and NK cells when stroke occurs, suggesting that IP-10 expression induces NK cell infiltration following cerebral ischemia (Figure 3I,J).

CXCR3 regulates leukocyte trafficking, and binding of chemokines such as IP-10 can induce various cellular responses [32]. Most notably, these changes can include integrin activation, cytoskeletal changes, and chemotactic migration. However, these responses are restored following the dephosphorylation of intracellular receptors and their subsequent recycling to the cell surface. In the present study, detection of mean fluorescence intensity was used to evaluate the action of IP-10 on NK cells. As a result, IP-10 was found to activate CXCR3 expression by NK cells (Figure 4A), and this effect was dose-dependent (Figure 4B). However, IFN-γ-mediated cytoxicity associated with IP-10-induced activation of NK cells was not observed (data not shown). Therefore, it appears that IP-10 is only a chemotactic cytokine for NK cells following cerebral ischemia.

NK cells respond to several chemotactic factors, including MIP-1 and Mig [33]. However, IP-10 is the primary chemotactic factor for NK cells in the brain. In the present study, IP-10 was found to be primarily secreted by neural cells rather than brain microvascular endothelial cells under OGD conditions (data not shown). In the brain, the stability of the microenvironment is maintained by BBB permeability [34]. Correspondingly, damage to the BBB can promote secondary inflammation injury following stroke [35]. By co-culturing neural cells and cerebral microvascular endothelial cells under OGD conditions as an analogue of ischemia and the BBB, an increase in permeability was observed following the addition of NK cells (Figure 5A). Furthermore, this increase in permeability, as well as the migration of NK cells, was reduced following neutralization of IP-10 (Figure 5B). These results strongly suggest that NK cells negatively affect the BBB during stroke, and IP-10 enhances the infiltration of NK cells through the BBB following cerebral ischemia.

## Conclusions

In summary, NK cells were found to participate in the early stages of stroke and to induce the necrosis of neural cells via IFN-γ. Based on these results, the capacity to neutralize IFN-γ represents an opportunity to stabilize the microenvironment associated with cerebral ischemia. In addition, NK cells were found to damage the BBB in response to IP-10 as a chemoattractant. These results suggest that the damage mediated by NK cells following stroke may be attenuated by neutralizing IP-10 and other factors that are attractive to NK cells, thereby preventing the release of cytotoxic cytokines. It is anticipated that further studies of these mechanisms may improve the indications of clinical therapy for brain ischemia. Furthermore, future studies will need to focus on NK cells present in ischemic tissue and their interactions with other immune cells during different phases of stroke, particularly interactions that lead to damage of the BBB.

## Abbreviations

TTC: 2,3,5-triphenyltetrazolium chloride; pMCAO: permanent middle cerebral artery occlusion; BBB: blood brain barrier; OGD: oxygen glucose deprivation; PBS: Phosphate Buffered Saline; HBSS: Hank’s Balanced Salt Solution; ANOVA: analysis of variance; DMEM: Dulbecco’s-modified Eagle’s medium; FBS: fetal bovine serum; OGD: oxygen-glucose deprivation; PI: propidium iodide.

## Competing interests

The authors declare that they have no competing interests.

## Authors’ contributions

YZ, ZG, GW and HL designed research, performed research, analyzed data, and wrote the manuscript. DW, TZ, BS, LM, JW, YL, QK, XL, YZ, HZ and JH performed research, analyzed data, and wrote the manuscript. All authors read and approved the final manuscript.
